# Distinguishing emotional distress from mental disorder in primary care: a qualitative exploration of the Four-Dimensional Symptom Questionnaire

**DOI:** 10.3399/BJGP.2023.0574

**Published:** 2024-06-11

**Authors:** Adam WA Geraghty, Sian Holt, Carolyn A Chew-Graham, Miriam Santer, Michael Moore, Tony Kendrick, Berend Terluin, Paul Little, Beth Stuart, Manoj Mistry, Al Richards, Debs Smith, Sonia Newman, Shanaya Rathod, Hannah Bowers, Harm van Marwijk

**Affiliations:** Primary Care, Population Sciences and Medical Education, University of Southampton, Southampton, UK.; Primary Care, Population Sciences and Medical Education, University of Southampton, Southampton, UK.; School of Medicine, Keele University, Keele, UK.; Primary Care, Population Sciences and Medical Education, University of Southampton, Southampton, UK.; Primary Care, Population Sciences and Medical Education, University of Southampton, Southampton, UK.; Primary Care, Population Sciences and Medical Education, University of Southampton, Southampton, UK.; Department of General Practice, Amsterdam University Medical Centre, Amsterdam, the Netherlands.; Primary Care, Population Sciences and Medical Education, University of Southampton, Southampton, UK.; Queen Mary University of London, London, UK.; Primary Care, Population Sciences and Medical Education, University of Southampton, Southampton, UK.; Primary Care, Population Sciences and Medical Education, University of Southampton, Southampton, UK.; Primary Care, Population Sciences and Medical Education, University of Southampton, Southampton, UK.; Primary Care, Population Sciences and Medical Education, University of Southampton, Southampton, UK.; Southern Health NHS Foundation Trust, Southampton, UK.; Primary Care, Population Sciences and Medical Education, University of Southampton, Southampton, UK.; Department of Primary Care and Public Health, Brighton and Sussex Medical School, Brighton, UK.

**Keywords:** distress, general practice, mental health, questionnaire

## Abstract

**Background:**

Primary care clinicians see people experiencing the full range of mental health problems. Determining when symptoms reflect disorder is complex. The Four-Dimensional Symptom Questionnaire (4DSQ) uniquely distinguishes general distress from depressive and anxiety disorders. It may support diagnostic conversations and targeting of treatment.

**Aim:**

To explore peoples’ experiences of completing the 4DSQ and their perceptions of their resulting score profile across distress, depression, anxiety, and physical symptoms.

**Design and setting:**

A qualitative study was conducted in the UK with people recruited from primary care and community settings.

**Method:**

Participants completed the 4DSQ then took part in semi-structured telephone interviews. They were interviewed about their experience of completing the 4DSQ, their perceptions of their scores across four dimensions, and the perceived utility if used with a clinician. Interviews were transcribed verbatim and data were analysed thematically.

**Results:**

Twenty-four interviews were conducted. Most participants found the 4DSQ easy to complete and reported that scores across the four dimensions aligned well with their symptom experience. Distinct scores for distress, depression, and anxiety appeared to support improved self-understanding. Some valued the opportunity to discuss their scores and provide relevant context. Many felt the use of the 4DSQ with clinicians would be helpful and likely to support treatment decisions, although some were concerned about time-limited consultations.

**Conclusion:**

Distinguishing general distress from depressive and anxiety disorders aligned well with people’s experience of symptoms. Use of the 4DSQ as part of mental health consultations may support targeting of treatment and personalisation of care.

## Introduction

In the UK, the majority of people’s mental health concerns will be managed by primary care clinicians from various backgrounds.^1^ A range of problems are presented to primary care, from people presenting with minor symptoms through to those presenting with severe mental illness.^2^ There is a tension that contributes to this complexity, with increasing suggestions of a mental health crisis in many areas,^3^^–^^5^ and lack of adequate services to respond, alongside parallel concerns about overmedicalisation and related overtreatment.^6^^,^^7^ Models for assessment also vary greatly, from diagnostic criteria-based approaches (for example, use of symptom questionnaires such as the Patient Health Questionnaire [PHQ]-9)^1^ to calls for alternative non-categorical strategies based on shared understanding^8^ (for example, prioritising key issues facing the individual, how they link, and developing a shared action plan). Consequently, clinicians use a wide range of differing models and treatment with patients who present with mental health concerns.^9^ This variation in practice raises questions about optimal care.

Dutch researchers have developed an approach for mental health assessment specifically designed for use in primary care.^10^^,^^11^ It involves a multidimensional assessment with patients’ symptoms reported and scored across four areas: distress, depression, anxiety, and physical symptoms. This is done with the use of the Four-Dimensional Symptom Questionnaire (4DSQ)^10^ before or between consultations. The resulting score profile across the dimensions is then used to inform decision making within the consultation. The 4DSQ has been validated in primary care samples,^10^^–^^12^ is available in 17 languages, and features in Dutch national guidelines for depression, where its use is recommended to support initial diagnostic conversations.^13^

What makes this approach particularly useful for the wide range of symptoms seen in primary care is the unique distinction between general distress and depressive and anxiety disorders.^12^ Importantly, the model that underlies the approach and questionnaire was developed directly from primary care patient symptom data.^10^ Distress is defined here as general negative, cognitive, and emotional symptoms that often stem from difficulties coping with stressors.^12^^,^^14^ Depression and anxiety are defined as specific disorders of emotion regulation.^10^^,^^12^ By separating distress from disorder, this approach keeps diagnostic labels, but importantly tightens their conceptualisation. Additionally, it provides space for symptomatically severe distress that may not be driven by ‘dysfunctioning processes’ whether psychological, developmental, or biological, underlying mental function: a central but often not discussed aspect of the American Psychiatric Association definition of mental disorder.^15^ It represents an approach developed from, and grounded in, primary care, differing from widely used alternative models that stem from psychiatry.^8^ Consequently, research on the 4DSQ provides the opportunity to explore the utility of primary care-driven notions of ‘depression’ and ‘anxiety’ and their relationship to more general distress, amid longstanding debates about *Diagnostic and Statistical Manual of Mental Disorders*^15^ definitions of these conditions.^16^

**Table:** How this fits in

A range of different approaches have been suggested to support primary care clinicians in the identification and management of mental health problems, from brief depression questionnaires, to approaches focusing on shared understanding within consultations. The Four-Dimensional Symptom Questionnaire (4DSQ) is a questionnaire developed in primary care that can support this process by distinguishing general distress from depressive or anxiety disorder. In this study we show that people recruited from primary care and community settings find completing a multidimensional questionnaire acceptable and find the splitting of general (potentially severe) distress from depression and anxiety helpful. Use of the 4DSQ may support collaborative diagnostic conversations as part of primary care consultations.

Using an approach that supports clinicians and patients to collaboratively discuss and distinguish between (potentially severe) distress and mental disorder may be beneficial. It may facilitate personalised care, and thus a more targeted approach to treatment, as well as identifying people who may benefit from preventive interventions. This approach could potentially reduce medicalisation of symptoms and unnecessary (and possibly harmful^6^^,^^17^^,^^18^) overtreatment with antidepressant medications. Finally, it may support wider ranging diagnostic conversations in the consultation, importantly including a domain often endorsed by patients when conceptualising their own symptoms, for example, distress/stress without depression.^19^^,^^20^

The 4DSQ is not widely used in the UK and it is a longer questionnaire than scales such as the PHQ-9, comprising 50 items. A critical step in understanding the potential of this approach in the UK is to determine if the questionnaire and the resulting multidimensional score profile is acceptable and perceived as useful by those who may consult in primary care regarding their mental health. Although there has been extensive quantitative psychometric evaluation of the 4DSQ,^11^^,^^21^^–^^23^ to our knowledge there have been no qualitative explorations of its use. Therefore, in this qualitative study, we aimed to:

- explore the acceptability of completion of the 4DSQ in a UK sample with diverse backgrounds; and
- explore people’s perceptions of an approach that distinguishes between distress and disorder.

## Method

### Design

We conducted a qualitative study using semi-structured interviews.

### Participants and recruitment

We kept eligibility broad for the qualitative study: participants were aged ≥18 years, with experience of stress or low mood, or identifying as experiencing common mental health problems such as depression and anxiety. Participants needed to be able to speak English, provide informed consent, and follow study procedures.

Two recruitment methods were used: recruiting through general practices and recruiting directly from the community. General practice recruitment involved list searches for mental health-related codes (for example, low mood, stress, worry, depression, and anxiety), with resulting lists screened for eligibility by practice staff. Those deemed eligible were sent a study information pack. Community recruitment involved using leaflets and posters given out at the community centre in Southampton and sent to our community links. We also recruited via the social media pages of the community centre we worked with. Interested potential community participants visited a website containing an online study pack and a route to contact the study coordinator. We sampled purposively, aiming to recruit people with a range of characteristics in terms of age, gender, ethnicity, and educational attainment.

### Data generation and interviews

Participants were recruited between June and December 2022. Participants provided consent and completed the Four-Dimensional Symptom Questionnaire (4DSQ,^10^ see Boxes 1 and 2) ahead of a semi-structured interview conducted over the phone. Participants could complete this online or on paper. If completed online, participants’ score profiles were available to the interviewer ahead of the interview. If completed on paper, the interviewer went through the participants’ scores on the 4DSQ over the phone, calculating the resulting score profile for discussion in the interview.

**Box 1.** Key features of the Four-Dimensional Symptom Questionnaire^a^

Number of scales: four (distress, depression, anxiety, physical symptoms) Number of items: 50 (distress: 16; depression: 6; anxiety: 12; physical symptoms: 16) Response options and scoring: no, 0; sometimes, 1; regularly, 2; often, 2; very often or constantly, 2 Recall period: the past week

**Box 2.** Details of the four dimensions of the Four-Dimensional Symptom Questionnaire including scoring^a^

**Scale**	**Low**	**Moderately high**	**Very high**
**Distress**	• 0–10: normal distress; in principle no action necessary	• 11–20: increased distress with the threat of dysfunctioning; stress reduction is desirable	• 21–32: severe distress with high risk of dysfunctioning (sick leave); stress reduction is indicated
**Depression**	• 0–2: probably no depressive disorder	• 3–5: possible depressive disorder; wait-and-see and re-evaluation after a few weeks; if indicated clinical depression diagnosis	• 6–12: relatively high risk of a depressive disorder; clinical depression diagnosis is indicated
**Anxiety**	• 0–3: probably no anxiety disorder	• 4–8: possible anxiety disorder; wait-and-see and re-evaluation after a few weeks; if indicated clinical anxiety diagnosis	• 9–24: relatively high risk of one or more anxiety disorders; clinical anxiety diagnosis is indicated
**Physical** **symptoms**	• 0–10: relatively normal bodily reaction to stress	• 11–20: possibly problematic physical symptoms with the threat of dysfunctioning; discuss with patient	• 21–32: high risk of problematic physical symptoms; discuss with patient, consider cognitive behavioural therapy or referral

a *Interpretation of scale scores based on English norms.*

The interview topic guide was developed by our team, including public contributors. The interviews were designed to explore participants’ mental health experiences broadly, before focusing on their experience of completing the 4DSQ (see Supplementary Information S1 for the interview schedule). Scores across the four dimensions were then fed back to them, along with an indication of whether their score on each dimension was low, moderately high, or very high. Participants were asked to reflect on their personal score profile in terms of accuracy, usefulness, and their more general perceptions.

All interviews were carried out by the second author, a non-clinical research fellow with a PhD in psychology. The interviewer had no relationship to the participants before the interviews. Interviews were stopped when we agreed we had sufficient^24^ information power^25^ from a diverse sample, enabling us to reach the analytic aims of the study. Interviews were audio-recorded and were transcribed verbatim by a professional transcription company. All participants were offered £40 in gift vouchers to recognise their contribution.

### Analysis

A thematic analytic approach drawing from elements of Joffe and Yardley^26^ and Braun and Clarke^27^ was led by the first author and involved the full team. All transcripts were read in detail and coded, attaching labels to meaning within the transcripts using NVivo (v11). The initial coding was developed into a large document of code names and examples with multiple data excerpts. This document was designed to facilitate access to data and coding structures for discussion with the full team including public contributors. Following ongoing discussion, analysis continued through constant comparison of codes and related data segments, with a particular focus on disconfirming evidence. Through this process, overarching, primarily descriptive themes were developed. The team then collaborated iteratively until a final set of themes was agreed on.

The team brought a range of experience to the developing analysis; our team comprised three academic psychologists, seven academic GPs, a psychiatrist, a public engagement expert, and three public contributors with diverse lived experience.

### Public involvement

The study was developed and conducted by working closely with our diverse public contributor team (comprising three members, the tenth, eleventh, and twelfth authors). The public contributors were a core part of the study management group that met monthly and guided all aspects of the project. They also contributed to analysis and write-up for publication. In addition, we developed a community outreach approach that involved multiple meetings with a group of adults at a Sure Start community centre from an underserved area in Southampton, meetings with ethnically diverse community leaders, and working with a group of older adults from the West Midlands organised through the Beth Johnson Foundation charity. In these meetings we discussed experiences of mental health symptoms and their management in primary care. These discussions informed study design and supported contextualisation of our findings. All the community groups we worked with felt there could be benefits to an approach where distress was acknowledged as related to, but distinct from, major depressive and anxiety disorders. Many provided examples from their community where they felt overuse of disorder terms had led to overtreatment. The opposite was also described, where people felt they had to push hard for acknowledgment of disorder and appropriate treatment in the context of complex life events. Many also described frustration with not being listened to within the medical system. When described to them, the groups felt that a 4DSQ approach had potential, if it was used as a way to listen more carefully to patients.

## Results

We recruited 24 participants into the qualitative study, 13 through community recruitment routes and 11 through general practices. See Table 1 for participant characteristics. Twenty participants completed the 4DSQ online and four completed the paper version of the questionnaire. The mean duration for the interviews was 53 min (range 27–79 min).

**Table 1.** Participant characteristics

**Characteristic**	**Value**
**Gender, *n***	
Female	15
Male	9

**Age, years, *n***	
18–27	2
28–37	8
38–47	5
48–57	5
58–67	2
68–77	2

**Highest educational qualification, *n***	
No formal qualification	1
GCSE or equivalent	3
A-level or equivalent	10
Degree	4
Masters	5
Doctoral degree	1

**Ethnicity, *n***	
White — English/Welsh/Scottish/Northern Irish/British	20
Asian/Asian British — Indian	2
Mixed/multiple ethnic groups — White and Asian	1
Black/African/Caribbean/Black British — Caribbean	1

**4SDQ dimension scores, mean (SD)**	
Distress	15.7 (8.4)
Depression	3.1 (2.8)
Anxiety	5.3 (5.0)
Physical symptoms	9.6 (6.3)

*4SDQ = Four-Dimensional Symptom Questionnaire. GCSE = General Certificate of Secondary Education.*

At the beginning of the interviews, participants gave wide-ranging descriptions of their mental health experiences; some people described experiences of diagnoses and medication, for others long histories of complex life events drove their narratives. The central analysis presented here was specifically on discussion of the 4DSQ and usefulness of the resulting symptom profiles. We developed six themes:

- general experiences of completing the 4DSQ;
- perspectives on specific dimensions;
- accuracy of overall 4DSQ symptom profile;
- breaking down symptoms for self-understanding;
- the importance of discussing symptoms; and
- supporting a broader understanding for clinicians.

Illustrative quotes are provided to support the thematic analysis, with participant identifiers given (C = community recruitment; PC = primary care recruitment), as well as gender and age.

### General experiences of completing the 4DSQ

Most participants did not report difficulties when completing the 4DSQ; reporting it took them between 5 and 15 min to complete. This finding was consistent across the varied educational backgrounds of the participants. As the 4DSQ is a 50-item questionnaire, participants were asked to reflect on its length and how they found completing it. Again, most participants did not perceive it to be a barrier. For some, the longer length was seen as a positive aspect of the 4SDQ, enabling the participants to provide more detail about their experience:

Interviewer: *‘How did you find completing it, how was that?’*

Participant: *‘Yes, fine. It was pretty good really because it was a little bit more in-depth. It was very to the point, and it was all the same answers that you could have given. It was all the same multiple choice; it wasn’t difficult to get your head around or anything like that. So you can do it quite easily.’*

(C005, female [F], 38 years)

Interviewer: *How did you get on with completing the questionnaire?*

Participant: *‘It was okay. I’m dyslexic, so sometimes questionnaires are a bit daunting, but where they were short-ish questions, it was fine. You didn’t need to read it multiple times.’*

(PC001-001, F, 30 years)

One participant did feel the questionnaire was too long, reporting concerns when continuing to answer questions beyond a certain point:

*‘I think it was quite long-winded. It was very long, and I started stressing out after question twenty-five.’*

(C003, F, 23 years)

### Perspectives on specific dimensions

Most reported that their score on the specific dimensions of the 4DSQ felt representative of their current experience. This was the case for the distress dimension, where participants often felt distress scores were appropriate:

Interviewer: [Referring to an elevated distress score] *‘What do you think about that?’*

Participant: *‘Yes, I would gauge that as being fairly accurate, really. I wouldn’t necessarily contest that. Yes, I get through my day-to-day activities, but yes. There’s always something, a little bit of nagging going on. So yes, I can understand that, I can relate to that.’*

(C031, male [M], 50 years)

Participant: *‘It just sounds like me. I don’t know. I do get distracted at work because of day-to-day stresses, yes. If it’s not work it’s …* [describes family issues].’

(PC004-008, M, 65 years)

Depression scores were also often seen as reflective of current experience. When higher depression scores were contrasted with moderate/low distress scores in the same symptom profile, the participants appeared to conceptualise depression as a persistent long-term condition:

Interviewer: *‘Your score for depression was what we’d put as moderately high. It shows you are possibly experiencing clinical depression but it’s obviously not into that very, very high level. What do you think about that?’*

Participant: *‘Yes, I’d say that’s exactly it. Clinical depression’s what I have had in the past and what I wake up in the morning to for no real reason. At the moment I’m — yes, I wake up in the morning and if I am blue I seem to have the tools to be able to get up and shake it off and get on with the day. It’s still there.’*

(PC002-001, F, 58 years)

The distinction between distress and depression enabled by the 4DSQ was supported by participants who had experienced depression previously. Some of these participants had a high distress score coupled with low depression score and felt that this was an accurate representation:

Interviewer: [Referring to a low depression score] *‘That shows you’re probably not experiencing a depressive disorder, based on what this questionnaire says. What do you think about that?’*

Participant: *‘Yes, I agree with that. I know what it feels like to feel things are meaningless and that there’s no point to anything. That’s still quite fresh in my memory and I’m very aware that that’s not what I’m feeling at the moment, and that’s not what I felt in the past seven days. It’s fresh in my memory.’*

(C010, F, 35 years)

Less frequently, participants expressed surprise that scores on some dimensions (for example, depression) were higher than they were expecting. In these cases, the higher scores on the particular dimension appeared to provide an opportunity for reflection on their situation:

Interviewer: [Referring to high depression scores] *‘What do you feel about that?’*

Participant: *‘Yes. It does surprise me a little because I keep thinking that maybe my anxiety and worry is worse, but actually when I sit back and I try to reflect, I see it. I think I don’t want to accept it lately.’*

(C007, F, 31 years)

### Accuracy of overall 4DSQ symptom profile

Towards the end of the interviews, participants were provided with a summary of their overall scores on the 4DSQ by the interviewer. For the majority, these were described as accurate and useful descriptions of how they were feeling:

Interviewer: *‘You had a very high stress score. Then, a moderately high depression and anxiety score and then a low related physical symptoms score. What do you think of that as an overall summary of how you felt during those seven days?’*

Participant: *‘I’d say that’s a fairly accurate picture of any given stressful period for me, I’d say. I’d say that’s definitely the hierarchy of how things look anyway. It is probably spot on. Yes.’*

(PC006-002, M, 32 years)

For one participant, the overall profile scores were surprising, leading them to question the accuracy of two dimensions:

Interviewer: *‘So our questionnaire’s showing a high stress score and then a moderate anxiety and depression score and then a low physical symptoms score. What do you think about that overall, as a summary?’*

Participant: *‘I’m not sure it is accurate. I mean the anxiety one doesn’t surprise me — I would have thought that would be moderate or high. The depression one I thought would be moderate to low, but the stress and the physical symptom ones are the ones that stand out as being inaccurate to me. Although maybe I’m being too kind to myself on the stress side! Maybe I’m more stressed than I realised.’*

(PC005-003, F, 35 years)

### Breaking down symptoms for self-understanding

When reflecting on the usefulness of the overall score profile, participants often described the benefits of having symptom experience broken down by the 4DSQ. Being able to ‘score high on one but not the other’ (C007) appeared to support a more fine-grained self-understanding:

Interviewer: *‘So that’s across stress, depression, anxiety, and the physical symptoms. A little bit lower on the anxiety than anything else, but scoring very high on all four of those. Overall, what do you think of that as a summary of your mental health at the moment?’*

Participant: *‘Yes. I think, like I said, I’ve been not wanting to accept it maybe because I’m feeling a bit reluctant to see a GP, but it does make sense to me when it’s staring me in the face like this. I find it very helpful to see it broken down like this, as well.* […] *Obviously, I’m high across the board. But it’s also given me more places to go and — when it’s broken down like that, I can go and try and get to the cause maybe a bit easier.’*

(C007, F, 31 years)

*‘When I sit down and think about it, it seems to tally with how I feel, because I did worry that I was feeling quite down the last couple of days, but actually, like I said, I think that is more of an overwhelmed, stressed, anxious thing, which I think makes a lot more sense.’*

(C017, F, 35 years)

In one participant, the breakdown of the scores provided an opportunity for clarification. Although this participant scored high on distress and depression, they highlighted that stress was the key driver of their symptoms:

Interviewer: *‘So you’re scoring very high on distress, very high on depression, and low on anxiety and low on physical symptoms. What do you think of that as an overall picture, of what’s going on with you right now?’*

Participant: *‘Yes, I think it is definitely right. It is mostly the stress and I think that’s what, it’s what I’d like to point out a little bit more, is that it is more stress and it’s not necessarily things that can be or need to be treated with medication and things like that. So that was a better questionnaire and that to do and I think that’s more of what the doctors need to be using really, because I just think it’s a little bit more accurate on what is actually going on.’*

(C005, F, 38 years)

### The importance of discussing symptoms

The 4DSQ is designed to be used as part of a conversation with clinicians. Although the conversation in this study was with a non-clinical researcher, some participants highlighted the benefits of being able to talk through their symptom profile scores. This appeared to support both general validation of their symptoms, alongside the opportunity for adjustment and contextualisation:

*‘ Talking to you and going through things, makes me feel that I’m, sort of, I’m okay. So knowing I’m okay makes me go on a different path to the person who thought he had problems.’*

(PC005-001, M, 72 years)

*‘ So yes, I think it’s been really helpful, actually. I’m just slightly surprised, in a way, because sometimes I find these questionnaires can be a bit impersonal, but I think having the opportunity to talk it through has been quite useful … and also good to know I can think about it and adjust it if I need to, in a sense. So I can say, “Oh, well, actually, no, that doesn’t feel right for me.” I think knowing I can say that makes me feel a bit more involved in the process, or that I can engage a little bit more with the process.’*

(C017, F, 35 years)

The below participant again highlights the usefulness of a dynamic process where scores are part of a conversation. They also describe their experience of the 4DSQ as being better than previous questionnaires that they found difficult to understand:

*‘ Yes, I think so, because I think it summarises the conversations that we’ve had within a relatively short space of time, and if nothing else, it starts the conversation, doesn’t it? It just starts a conversation. It’s a lot better than the one I did, because the one I did, I don’t even remember, I think it’s a scale of five, and they’d say, “Well, what would you be?” Like, “What? I don’t really understand”, but yes, it’s a relatively easy way, and it can be done really quickly.’*

(C032, M, 41 years)

### Supporting a broader understanding for clinicians

Participants were asked to reflect on the potential usefulness of using the 4DSQ with clinicians. Here, participants often discussed the benefits of the greater detail provided by the multiple dimensions. They discussed how that might help identify causes and facilitate more appropriate treatment:

*‘ I think the better understanding the person that’s diagnosing and helping you has, the more help you’re going to get that’s right for you, rather than it being more generalised everyone gets the same treatment.’*

(PC001-001, F, 30 years)

*‘ I just think it kind of opens up for more questions, because then you can just, more pinpoint around, so the stress one and then you could talk more about how to deal with that or what is actually going on and it would help me and it would help other people in the same boat, to talk more about what is actually going on, and to get the help that way, definitely.’*

(C005, F, 38 years)

Some participants were concerned about whether the contextualisation and full conversation, as discussed, could be achieved in current day-to-day primary care practice. There was concern that, without appropriate time, people may not feel adequately heard:

*‘ My only concern sometimes with these questionnaires is that they’re not contextualised properly, and sometimes people, I think, can feel then slightly dismissed. So I think that’s a danger with it, but I think if there’s a proper conversation happening around it, then it could be really helpful.’*

(C017, F, 35 years)

*‘I just couldn’t see a GP having that amount of time to go through it. I guess if you’re comfortable enough with your GP and they’ve got the time to go through it properly with you, and you felt like you were being heard, and that it was going to be, I guess, this is going to be a helpful tool for a next step, then, I think it would be a really good thing.’*

(PC006-002, M, 32 years)

## Discussion

### Summary

The 4DSQ is a primary care-centric questionnaire that uniquely splits general distress from depressive and anxiety disorder. In this qualitative study, participants found the 4DSQ easy to complete and, for most, the length of the questionnaire was not a barrier to use. The resulting score profile across the four dimensions was generally found to correspond well with symptom experience, and the separate scores for dimensions (for example, distress and depression) appeared to support greater self-understanding. Some participants valued conversations about their score profile, allowing for clarification and contextualisation. Most believed using the 4DSQ with clinicians would be helpful, supporting improved treatment decisions and shared understanding. There was concern from a small number of participants about whether primary care clinicians would have time to have the appropriate conversations to make use of the person’s 4DSQ symptom profile.

### Strengths and limitations

The strengths of this study include strong public contributor collaboration throughout, from study application to co-authorship of this paper. This study and analyses were informed by ongoing community outreach work with diverse groups who were unlikely to become involved in more formal public involvement and engagement roles. We used community and primary care recruitment routes to recruit participants who may not respond to formal research invites from their GP surgeries. Regarding study limitations, we struggled to recruit more participants from ethnically diverse backgrounds, despite trying different community recruitment approaches. Through our community outreach work we learnt that this was likely because of cultural concerns with both the mental health topic and some of the formal requirements for taking part in interviews (for example, consenting to recorded interviews).

### Comparison with existing literature

Our finding that most participants appeared to value completing and discussing their score profile on the 4DSQ aligns with previous research focusing on different questionnaires used for assessing severity of depression.^28^^,^^29^ Patients have consistently reported valuing the use of questionnaires such as the PHQ-9 in depression management specifically, focusing on support for targeting of treatment and self-understanding.^28^ Clinicians’ views on depression questionnaires have been more mixed, with concerns about impact on the consultation, validity, and effect on clinician autonomy.^29^ There has yet to be research on clinicians’ experiences with a questionnaire that provides distinct distress and depression profiles for patients.

Although briefer questionnaires such as the PHQ-9 are designed to determine severity within one area/dimension, namely ‘depression’,^30^ the 4DSQ appeared to support participants’ self-understanding in discriminating between symptom categories. This discriminating function may be helpful in supporting clinical conversations; previous research suggests that people will often consult in primary care to find out if they are experiencing symptoms reflecting disorder, for example, ‘Am I depressed?’^19^ Participants discussed the value of a conversation about their 4DSQ scores, but there was also concern about whether this is possible in day-to-day, time-pressed consultations. This finding echoes research suggesting the importance of using questionnaires in a ‘relational’ way,^31^ where the clinician and patient can discuss scores and their meaning in the context of the patients’ lives. Working in such a relational way with the resulting score profile from the 4DSQ may be a key factor in whether it supports or detracts from mental health-focused consultations.^32^

### Implications for research and practice

The 4DSQ is recommended for use in Dutch national guidelines and a previous Dutch trial has shown its positive impact on mental health symptoms.^33^ However, research is needed to determine whether use would support positive outcomes in a UK primary care context. A critical next step for researchers is to explore the experiences of clinicians using the 4DSQ in practice. Working closely with clinicians will be important to ensure problems they reported with earlier depression questionnaires are avoided, as well as supporting integration into information technology systems and clinician workflows. It will also be important to collaborate closely with people from diverse backgrounds in developing systems to support access and completion of the 4DSQ, including accessible score profile descriptions. In this way, processes that include the 4DSQ can be as inclusive as possible.

More broadly, if the 4DSQ can be implemented in a way that is deemed helpful by clinicians and patients, it may have large implications for practice. Supporting a process to collaboratively distinguish between distress and disorder could have a direct impact on care provision, including medication prescriptions (for example, initiation and repeat prescribing of antidepressants) and referrals to psychological services. Where the 4DSQ shows high distress without likely depressive disorder (a score profile that is common in primary care^14^) alternatives to depression treatment may need to be considered, and more suitable care options developed.

To conclude, an educationally diverse UK sample described the 4DSQ as straightforward to complete and found the resulting symptom profile aligned well with their own symptom experience. Distinguishing distress from disorder was perceived as useful, for both supporting self-understanding and potentially improving conversations with clinicians.
